# Missed Opportunities for Lung Cancer Screening Among Patients With Behavioral Health Disorders With Elevated Cigarette Smoking Rates: Lung Cancer Screening and Behavioral Health

**DOI:** 10.1016/j.chest.2024.11.039

**Published:** 2024-12-12

**Authors:** Anastasia Rogova, Lisa M. Lowenstein, Lorraine R. Reitzel, Kathleen Casey, Robert J. Volk

**Affiliations:** a Department of Health Services Research, The University of Texas MD Anderson Cancer Center, Houston, TX; b Department of Behavioral Science, The University of Texas MD Anderson Cancer Center, Houston, TX; c Integral Care, Austin, TX

**Keywords:** behavioral health, cancer prevention, lung cancer screening

## Abstract

Annual lung cancer screening using low-dose CT (LDCT) imaging effectively reduces mortality from lung cancer and is recommended for people who are at high risk of developing the disease. The utilization of lung cancer screening, however, has remained low. Due to significantly higher cigarette smoking rates, patients with behavioral health disorders (those living with mental illness and/or substance use disorders) are more likely to be diagnosed with and die of lung cancer; at the same time, they are less likely to undergo cancer screenings. There is an urgent need for targeted efforts to improve access to lung cancer screening among this population disproportionately affected by the disease. In this commentary, we propose integrating lung cancer screening facilitation into services provided by behavioral health professionals who are uniquely positioned to reach these patients and deliver interventions to increase uptake of cancer screenings. We suggest several measures that could improve lung health outcomes of patients with behavioral health disorders: (1) training behavioral health professionals in lung cancer screening eligibility assessment; (2) providing patients with educational materials; (3) integrating shared decision-making counseling for lung cancer screening into behavioral health care settings; (4) providing the practical support needed to access screening; and (5) establishing effective partnerships with community organizations. Regardless of the level of engagement, possibly ranging from brief training to the implementation of comprehensive programs, any involvement will benefit patients. This integrated approach will contribute to reducing lung cancer mortality among patients with behavioral health disorders who have long experienced systemic health inequities.

Lung cancer is the leading cause of cancer-related mortality in the United States, contributing to an estimated 127,070 deaths in 2023.^1^ Earlier diagnosis has the potential to improve survival rates from < 10% for metastasized cancer to > 50% for localized disease.^2^ The National Lung Screening Trial found that annual low-dose CT (LDCT) lung cancer screening reduces overall lung cancer mortality by 16% to 20%.^3^ The US Preventive Services Task Force recommends annual screenings using LDCT imaging for eligible individuals,^4^ as these screenings provide an opportunity to diagnose lung cancer at an earlier and more treatable stage. Despite these proven benefits, the utilization rates have persistently remained low.^5^, ^6^, ^7^ In this commentary, we propose integrating lung cancer screening facilitation into behavioral health services and outline strategies to improve screening uptake and outcomes for patients with behavioral health disorders.

## Patients With Behavioral Health Disorders Have Elevated Rates of Cigarette Smoking and Higher Nicotine Dependence

Tobacco use presents a health disparity issue because it is not equally distributed among all population groups in the United States.^8^ Notably, people living with behavioral health disorders (mental health and/or non-nicotine substance use disorders) smoke cigarettes at significantly higher rates than the general public. Although only 11.5% of the general population of the United States smoked cigarettes in 2020,^9^ up to 50% of people living with behavioral health disorders engaged in smoking.^10^ This inequity is rooted in multiple interrelated factors, including neurobiological mechanisms that heighten vulnerability to tobacco addiction among patients with psychiatric disorders,^11^ as well as psychosocial and environmental factors that increase their exposure to and uptake of tobacco.^12^ People with behavioral health disorders have long used tobacco in an attempt to alleviate their symptoms,^13^ and cigarette smoking as a form of self-medication has been further perpetuated by tobacco companies promoting cigarettes as a stress management tool, purportedly improving mental health conditions such as depression, anxiety, and mood disorders.^14^^,^^15^ Historical evidence reveals that the tobacco industry has been involved in efforts to oppose smoking bans in psychiatric hospitals; moreover, they were actively supplying cigarettes to patients in psychiatric settings, perpetuating a culture of smoking within these institutions.^14^

Even though a sizeable body of evidence now suggests that quitting smoking leads to better mental health and improved outcomes of substance use treatment, misconceptions that linked cigarette smoking to improved mental health and sobriety are still widely circulated among patients and behavioral health professionals.^16^ The impact of promoting tobacco use for self-medication purposes and avoiding nicotine dependence care within the behavioral health community has had long-lasting effects. Patients with behavioral health disorders not only smoke at higher rates, but they also use more cigarettes than individuals in the general population who smoke. Nicotine dependence is higher among adults with behavioral health disorders, especially among those who are aged ≥ 50 years, which makes them even less likely to quit and further increases the risk of developing and dying of lung cancer.^10^^,^^17^ These individuals are often interested in quitting smoking, and their quit attempt rates are similar to other adults who smoke cigarettes; however, their success rates are much lower.^18^ In addition to having higher nicotine dependence, individuals with behavioral health disorders often face greater barriers in accessing evidence-based smoking cessation treatment.^19^^,^^20^ As a result, the existing data suggest that disparities in cigarette use among patients with behavioral health disorders continue to increase relative to the general population.^20^

## Patients With Behavioral Health Disorders Are More Likely to Be Eligible for Lung Cancer Screening

Higher smoking rates and levels of nicotine dependence make patients with behavioral health disorders more likely to meet eligibility criteria for lung cancer screening than people in the general population.^10^^,^^13^ For instance, one-third of patients aged ≥ 65 years with schizophrenia are eligible for lung cancer screening compared with only 13% of the older adult population in the United States.^21^ At the same time, individuals with behavioral health conditions are less likely to receive screenings for any type of cancer compared with the general population.^22^, ^23^, ^24^, ^25^ Although data on lung cancer screening among patients with behavioral health conditions are limited, these individuals are likely to face barriers to lung cancer screening similar to other types of cancer.^25^ As a result, patients with behavioral health conditions are more likely to be diagnosed with more advanced-stage cancer.^26^, ^27^, ^28^ For example, adults with schizophrenia are more than twice as likely to die of lung cancer compared with the general population.^29^^,^^30^ Despite this evident disparity in screening rates, a Cochrane review found no trials of interventions targeted at encouraging cancer screening uptake among adults with mental illness.^31^

## Patients With Behavioral Health Disorders Often Have Limited Contact With Health Care Providers

Studies have emphasized the importance of identifying communities that are at the greatest risk for lung cancer while also being significantly disadvantaged in their ability to access primary care and lung cancer screening. For example, a report by the Equitable Implementation of Lung Cancer Interest Group within the Cancer Prevention and Control Research Network highlights people with mental illness as one of the priority communities for targeted efforts to improve lung cancer screening uptake.^32^ The health disparities experienced by patients with behavioral health disorders are exacerbated by a strong link between these conditions and social inequalities stemming from racial, ethnic, and environmental characteristics (eg, poverty, rural status).^33^

In addition to having disproportionately high rates of smoking, patients with behavioral health disorders often have limited access to primary and preventive health care, including smoking cessation and other screenings.^21^^,^^26^^,^^27^^,^^34^ These patients are less likely to have and regularly see primary care clinicians, and they generally underuse primary care.^23^^,^^35^, ^36^, ^37^, ^38^ For example, for many patients with serious mental illness, the only physician they routinely see is a psychiatrist, and behavioral health clinics are their only regular contact with the health care system.^35^^,^^39^ This fact is critical to consider when we address cancer screenings, as they are normally delivered as a part of primary care.

To alleviate health disparities experienced by this population, there have been calls to decrease fragmentation between behavioral health care and general medical care and emphasize potential benefits of integrated care models.^40^ However, despite some progress, high-quality fully integrated care is still not accessible to most individuals living with behavioral health conditions, and further efforts in designing care models that would improve physical and behavioral health care are needed.^39^^,^^40^ The American Psychiatric Association previously endorsed the need to enhance the role of psychiatrists in integrating behavioral and physical care and in assessing their patients’ physical health and diagnosing and treating them to reduce physical health disparities in patients with mental illness.^35^ Behavioral health providers should also be involved in lung cancer screening implementation to improve its uptake by their patients.^32^^,^^41^ Following this argument, we want to emphasize the importance of taking advantage of this missed opportunity to facilitate access to lung cancer screening services in behavioral health care settings.

## Behavioral Health Professionals Are Uniquely Positioned to Support Lung Cancer Screening

Providers who deliver behavioral health services include a diverse group of professionals and paraprofessionals, who have different education, training, and licensure requirements. These providers include psychiatrists, psychologists, psychiatric advanced nurse practitioners, addiction and substance abuse counselors, mental health counselors and therapists, peer support advocates, and others, with the total number of behavioral health specialists in the United States estimated to be > 700,000.^42^ Since the Centers for Medicare & Medicaid Services (CMS) requirements were updated in 2022, any health care professional is allowed to facilitate a shared decision-making (SDM) visit for lung cancer screening.^43^ Serving as a crucial, and often the only, point of contact with the health care system, behavioral health professionals establish a heightened level of trust with their patients, fostering effective communication and collaborative relationships. This is particularly important given that barriers to cancer screening among patients with behavioral health disorders extend beyond access to health care. There are multiple other factors that explain lower rates of screenings, including stigmatization, limited knowledge, and negative attitudes toward mental illness and substance use disorders within the health care system.^44^, ^45^, ^46^, ^47^

Health care professionals who normally manage screenings may have limited training needed to engage effectively with patients who live with behavioral health disorders and address their concerns. Behavioral health professionals, intimately familiar with their patients’ needs, are better equipped to engage them in SDM conversations and decision counseling within established trusting relationships. Prior research shows that trust in the referring clinician is particularly important for patients who decide to get screened for lung cancer.^48^ Behavioral health professionals are also better prepared to address issues related to potential stigmatizing attitudes, especially concerning continued tobacco use in the context of lung cancer screenings. Many of them are trained in trauma-informed care and motivational interviewing techniques, and both these approaches might be used to increase patients’ resilience and resolve their ambivalence and motivate them toward action. Leveraging the specialized training and unique patient relationships of behavioral health professionals is essential to ensuring that lung cancer screening services are accessible and well received by these patients.

## Lung Cancer Screening Services in Behavioral Health Care Settings

Cancer screenings, including those for lung cancer, are currently very rarely addressed in behavioral health settings.^35^^,^^49^ For example, a survey of mental health clinics conducted in New York State in 2018 showed that < 6% of clinics who participated in the study ensured that their patients were up-to-date with their cancer screening (including breast, colorectal, cervical, and lung; lung cancer screening was the lowest at 4.1%).^35^ Despite these dismal numbers, the existing data, although limited, suggest that behavioral health care providers could deliver interventions to increase uptake of cancer screenings among individuals with mental illness and substance use disorders; the same data show that these interventions could effectively occur in settings where patients receive behavioral health services.^21^^,^^50^ Prior studies also show that behavioral health care settings are able to integrate some preventive services into their routine clinical work. For example, the delivery of evidence-based tobacco cessation services have significantly increased in behavioral health care settings over the recent years, even though it is still not sufficient, and further efforts are needed in this area.^16^^,^^19^^,^^51^^,^^52^

There is an evident need to design multilevel interventions to facilitate access to lung cancer screening among eligible patients. We recommend several measures that would improve preventive care delivery in behavioral health care settings:

1. **Train Behavioral Health Specialists in Lung Cancer Screening Eligibility.** We recommend that, at a minimum, behavioral health specialists should receive training in lung cancer screening eligibility. This training will equip them with the knowledge to identify eligible patients and initiate informed conversations about lung cancer screening with their patients. Awareness of recommendations for lung cancer screening among providers is associated with an increased uptake of screening in various settings.^53^^,^^54^ Limited knowledge of lung cancer screening has been frequently reported both among health care providers and eligible individuals.^55^, ^56^, ^57^, ^58^ This lack of awareness presents an important barrier that has to be addressed as a crucial first step in improving access to screening.^59^ This provider training should become the first step of a comprehensive approach to lung cancer screening in behavioral health care settings. Considering the high rates of staff turnover in these settings,^60^ such trainings should take place regularly and be incorporated into annual and new employee training programs together with general tobacco cessation education.
2. **Provide Behavioral Health Settings With Patient Education Materials and Decision Aids.** This measure is particularly important for low-resource settings in which implementation of more comprehensive programs is not feasible, but these materials can be distributed among patients to inform them about lung cancer screening availability. Although it will not replace a consultation with a health care provider, high-quality decision aids will ensure that patients have access to correct information about screening options, their benefits, and their risks. It is also important to investigate the need for tailored materials for patients with behavioral health conditions who might have specific needs in terms of content, language, literacy levels, and visual presentation of the information. Prior research revealed that efforts to communicate lung cancer risks and benefits of screening often use stigmatizing language and imagery.^61^^,^^62^ The use of tailored materials employing culturally sensitive and empathic messaging is particularly important in discussions about lung cancer screening to minimize potential stigmatization of at-risk populations.^61^ Prior research has shown the effectiveness of using tailored patient-facing materials to inform eligible people about lung cancer screening.^63^, ^64^, ^65^ Involving patients in the design of these materials is another effective strategy to ensure their meaningful and positive engagement.^66^
3. **Integrate SDM Counseling for Lung Cancer Screening Into Behavioral Health Settings.** SDM is recommended prior to lung cancer screening by the US Preventive Services Task Force and is reimbursed by the CMS for people who meet beneficiary eligibility criteria.^43^ SDM is an opportunity for patients to discuss screening with a health care professional and receive support to make an informed decision about the screening. We suggest that SDM counseling can be successfully integrated into behavioral health care, although we anticipate that behavioral health care professionals might face barriers similar to those often reported by primary care providers, including lack of time and competing demands of their clinical practice.^67^ According to the CMS requirements updated in 2022, SDM counseling can be conducted by any health care professional who does not have to be a physician or a nonphysician practitioner but could be, for example, a nurse navigator, counselor, case manager, or peer support specialist.^43^ There are several studies that discuss the opportunities of delivering SDM counseling by different types of health professionals in various settings.^21^^,^^68^^,^^69^ Appropriate training and the use of high-quality decision aids ensure that the quality of SDM is maintained when delivered by these professionals.^70^
4. **Provide Practical Support to Patients to Improve Access to Screening.** It is equally important that patients who choose to screen for lung cancer receive practical assistance, which should include streamlining screening orders, providing support to schedule and complete screenings, and navigating patients to further diagnostic testing and treatment if needed. Patient navigation is proven to be effective to increase adherence to recommended cancer screenings, including but not limited to lung cancer.^71^ Access to a navigation program is even more important for individuals with behavioral health conditions who do not have regular contact with primary health care providers and who might experience extreme difficulties navigating a complex health care system.^72^ Willingness to screen annually and undergo treatment in case of cancer diagnosis are some of the important questions that must be considered when making decisions about lung cancer screening. Lack of support leading to patients’ inability to undergo diagnostic testing and treatment will minimize the benefits of screening.
5. **Develop Partnerships With Community-Based Organizations.** Establishing partnerships between behavioral health treatment centers and community-based organizations can address some of the barriers that might otherwise prevent the implementation of high-quality comprehensive lung cancer screening care. By bringing together strengths and resources of behavioral health professionals and community-based organizations, these partnerships can offer significant advantages and improve care. For instance, many behavioral health care centers do not have a physician on site who could place an order for LDCT imaging. These centers would benefit from partnering with primary care clinics to create a pathway by which behavioral health professionals can connect their patients with providers who can place LDCT imaging orders for patients who received SDM counseling and are interested in screening. In addition, partnerships with non-profit organizations or local government health initiatives can provide resources to offset the cost of screening for uninsured and underinsured patients. Effective partnerships might improve access to screening and reduce pressure on smaller, underresourced behavioral health care settings.

## Conclusions

Behavioral health care settings vary significantly in their capacity to provide preventive services, including available resources and the ability to connect patients with primary and specialty health care. We recognize that not all the measures will be feasible to implement in all settings. However, regardless of the extent of engagement in lung cancer screening care within behavioral health care settings (possibly ranging from brief training to the implementation of comprehensive programs), any level of participation will be valuable for patients who might lack information on screening availability, have limited access to the procedure, and who have long experienced systemic health disparities.

## Funding/Support

This work was supported in part by a fellowship award from 10.13039/100007313The University of Texas MD Anderson Cancer Center, Halliburton Employees Fellowship in Cancer Prevention Fund; the annual distributions of the Permanent Health Fund endowment received by the University of Texas MD Anderson Cancer Center from the state legislature; the Decision Science Core Facility supported in part by The University of Texas MD Anderson Cancer Center and the National Cancer Institute (P30CA016672) and grants from the 10.13039/100004917Cancer Prevention and Research Institute of Texas (RP230213 and PP230002).

## Financial/Nonfinancial Disclosures

None declared.
